# Outcomes and risk factors for delayed-onset postoperative respiratory failure: a multi-center case-control study by the University of California Critical Care Research Collaborative (UC^3^RC)

**DOI:** 10.1186/s12871-022-01681-x

**Published:** 2022-05-14

**Authors:** Jacqueline C. Stocking, Christiana Drake, J. Matthew Aldrich, Michael K. Ong, Alpesh Amin, Rebecca A. Marmor, Laura Godat, Maxime Cannesson, Michael A. Gropper, Patrick S. Romano, Christian Sandrock, Christian Bime, Ivo Abraham, Garth H. Utter

**Affiliations:** 1 Department of Internal Medicine, Division of Pulmonary, Critical Care and Sleep Medicine, University of California Davis, 4150 V Street, Suite 3400, Sacramento, CA 95817 USA; 2 Department of Statistics, University of California Davis, Davis, CA USA; 3 Department of Anesthesia and Perioperative Care, University of California San Francisco, San Francisco, CA USA; 4 Department of Medicine, University of California Los Angeles, Los Angeles, CA USA; 5 VA Greater Los Angeles Healthcare System, Los Angeles, CA USA; 6 Department of Medicine, University of California Irvine, Irvine, CA USA; 7 Department of Surgery, University of California San Diego, San Diego, CA USA; 8 Department of Anesthesiology and Perioperative Medicine, University of California Los Angeles, Los Angeles, CA USA; 9 Center for Healthcare Policy and Research, University of California Davis, Sacramento, CA USA; 10 College of Medicine, University of Arizona Health Sciences, Tucson, AZ USA; 11 Center for Health Outcomes and PharmacoEconomic Research, University of Arizona, Tucson, AZ USA; 12 Department of Surgery, Outcomes Research Group, University of California Davis, Sacramento, CA USA

**Keywords:** Respiratory failure, Postoperative, Risk factors, Surgical outcomes, Elective surgery, Matched case-control study, AHRQ PSI 11

## Abstract

**Background:**

Few interventions are known to reduce the incidence of respiratory failure that occurs following elective surgery (postoperative respiratory failure; PRF). We previously reported risk factors associated with PRF that occurs within the first 5 days after elective surgery (early PRF; E-PRF); however, PRF that occurs six or more days after elective surgery (late PRF; L-PRF) likely represents a different entity. We hypothesized that L-PRF would be associated with worse outcomes and different risk factors than E-PRF.

**Methods:**

This was a retrospective matched case-control study of 59,073 consecutive adult patients admitted for elective non-cardiac and non-pulmonary surgical procedures at one of five University of California academic medical centers between October 2012 and September 2015. We identified patients with L-PRF, confirmed by surgeon and intensivist subject matter expert review, and matched them 1:1 to patients who did not develop PRF (No-PRF) based on hospital, age, and surgical procedure. We then analyzed risk factors and outcomes associated with L-PRF compared to E-PRF and No-PRF.

**Results:**

Among 95 patients with L-PRF, 50.5% were female, 71.6% white, 27.4% Hispanic, and 53.7% Medicare recipients; the median age was 63 years (IQR 56, 70). Compared to 95 matched patients with No-PRF and 319 patients who developed E-PRF, L-PRF was associated with higher morbidity and mortality, longer hospital and intensive care unit length of stay, and increased costs. Compared to No-PRF, factors associated with L-PRF included: preexisiting neurologic disease (OR 4.36, 95% CI 1.81–10.46), anesthesia duration per hour (OR 1.22, 95% CI 1.04–1.44), and maximum intraoperative peak inspiratory pressure per cm H_2_0 (OR 1.14, 95% CI 1.06–1.22).

**Conclusions:**

We identified that pre-existing neurologic disease, longer duration of anesthesia, and greater maximum intraoperative peak inspiratory pressures were associated with respiratory failure that developed six or more days after elective surgery in adult patients (L-PRF). Interventions targeting these factors may be worthy of future evaluation.

**Supplementary Information:**

The online version contains supplementary material available at 10.1186/s12871-022-01681-x.

## Background

Postoperative respiratory failure (PRF) is a significant source of increased hospital length of stay, in-hospital and post-discharge morbidity, and in-hospital and long-term mortality; translating into markedly increased costs [1–5]. However, it is thought to be a potentially preventable adverse event [6] that is amenable to appropriate care [7]. With the volume of surgical procedures increasing annually, [8] there is an urgent and unmet need to reduce the incidence of PRF [9] by elucidating vulnerable PRF subpopulations by phenotypic presentation to identify: 1) which patients are most at risk, 2) which pathways contribute to increased morbidity and mortality, and 3) which patients are most likely to benefit from targeted preventive and therapeutic interventions [10].

The disparate burden of PRF by phenotype in surgical patients remains understudied. Also underreported, are differences between patients who develop PRF early versus late in their postoperative course. While there is no universal consensus on when respiratory failure is related to the surgical procedure, most clinicians, including our co-authors who are practicing anesthesiologists, surgeons, and critical care intensivists in the largest healthcare system in the state of California, describe the acute postoperative phase as lasting two to 3 days, and state that 5 days is a very reasonable cutoff that is clinically relevant when studying early postoperative complications. This five-day cutoff falls within the 0–7-day range described in four publications [11–14]. Among these publications is a study in which the authors used data from 4366 patients, 113 of whom developed early postoperative acute lung injury (ALI) or acute respiratory distress syndrome (ARDS), defined as occurring during postoperative day (POD) 0–5, to develop the Surgical Lung Injury Prediction Model [14]. The Surgical Lung Injury Prediction Model uses readily available preoperative risk factors to predict risk of early postoperative ALI or ARDS with high accuracy. The three additional studies used POD 0–3 [13], POD 0–5 [12], and POD 0–7 [11] to define the outcome of PRF, ALI, or ARDS. The dynamic nature of the postoperative timeframe is affected by many pre-, intra-, and post-operative factors acting synergistically and it is challenging to determine when PRF is a direct complication of the surgical events or a later consequence of additional events that occur during hospitalization [15]. Yet, it is precisely this lack of consensus that has hindered efforts to identify phenotypes and clinical trajectories to determine modifiable risk factors and reduce the incidence of PRF. There is also little convergence of research on the risk factors associated with PRF. Different patient populations, combined with the low incidence of this rare event, have led to risk models with varying factors [15–18].

The University of California Critical Care Research Collaborative (UC^3^RC) previously reported on patient- and procedure-related risk factors associated with PRF that developed on or before postoperative day five (early PRF; E-PRF) for 319 patients [19]; however, cases that occur later likely represent a different population of patients. In this study we compare patient outcomes and patient- and procedure-related risk factors associated with PRF that developed on or after postoperative day six (late PRF; L-PRF) to patients who did not develop PRF (No-PRF). We also separately compared outcomes of the L-PRF patients to cases of E-PRF. We hypothesize that patients who develop L-PRF will have worse outcomes and a unique set of modifiable patient- and procedure-related risk factors.

## Methods

This multicenter study by the UC^3^RC was approved by the Institutional Review Boards at the University of California, Davis (lead site) and Irvine, Los Angeles, San Diego, and San Francisco (collaborating sites), all of whom waived the requirement for informed consent for participation in the study. We previously described our case-control study methods, [19] including the validity of case ascertainment [20]. This manuscript adheres to Strengthening the Reporting of Observational Studies in Epidemiology (STROBE) guidelines [21].

### Study design and setting

This was a retrospective matched case-control study of all eligible adult elective surgery discharges between October 1, 2012, and September 30, 2015, at the five University of California academic medical centers. The outcome of interest was the diagnosis of L-PRF. We selected all predictor variables based on literature review and clinical expertise. L-PRF cases, which developed six or more days after surgery, were first compared to the No-PRF cohort and then to the E-PRF cohort.

### Study population

We used hospital administrative data submitted to the University Healthsystem Consortium (UHC), now Vizient Inc., based on the Agency for Healthcare Research and Quality (AHRQ) algorithm for Patient Safety Indicator 11 (PSI 11), Postoperative Respiratory Failure Rate, [22] to identify at-risk patients and potential cases of L-PRF among all adult patients discharged following an elective surgical procedure from all five sites during the study period. Because we were interested in potentially preventable cases of PRF, we excluded patients who required an open thoracic procedure (e.g., esophageal resection, lung cancer resection, open heart surgery).

#### Ascertainment of cases

Among 59,073 consecutive adult patients admitted for elective non-cardiac and non-pulmonary surgical procedures, 437 possible cases of PRF were identified based on administrative data. Each of these possible cases was then reviewed to confirm presence of at least one of the following criteria: [20, 23].

1. arterial oxygen partial pressure (PaO2) < 60 mmHg on room air; a ratio of arterial oxygen partial pressure (PaO2) to the fractional inspired oxygen (FiO2) < 300 [24]; or
2. physician documentation of PRF (due to hypoxemia or hypercarbia) or acute respiratory distress syndrome (ARDS); or
3. physician documentation of one of the following procedures because of respiratory compromise, insufficiency, or failure: [22]

- unplanned postoperative endotracheal re-intubation; or
- continuous mechanical ventilation (MV) for > 48 hours.

**Fig. 1:**
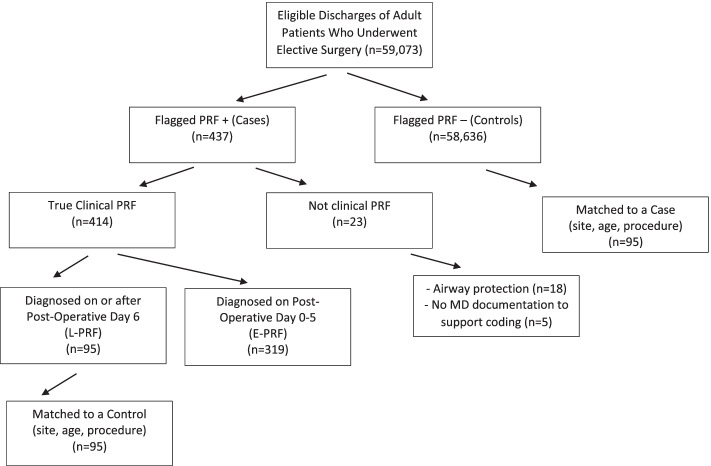
Ascertainment of Cases and Controls

We omitted 23 false positive cases and confirmed 319 cases of E-PRF and 95 cases of L-PRF (Fig. 1).

### Matching and verification of no-PRF

We matched L-PRF cases to No-PRF in a 1:1 ratio, randomly selecting the control(s) within strata based on age (by decade) (Additional file 1, Table S1), hospital (Additional file 2, Table S2), and principal ICD-9-CM procedure code (grouped by anatomic region and open versus minimally invasive approach using the Healthcare Cost and Utilization Project Clinical Classification Tools and Software [25]; (Additional files 3 and 4, Table S3 and Table S4). Once matched, each of the flagged No-PRF cases was reviewed to confirm the absence of true clinical PRF using the criteria defined above. This control group is referred to as No-PRF.

### Sample size and power analysis

A priori, using methods described by Dupont and Plummer, [26–28] we determined the odds ratio we would be able to detect for a sample size of 95 cases matched 1:1 to No-PRF. We calculated we would be able to detect true odds ratios more extreme than 0.25 or 2.81 in exposed relative to unexposed subjects with power of 80% at an α level of 0.05, assuming a probability of exposure among No-PRF of 20% and a correlation coefficient for exposure between matched cases and No-PRF of 0.2.

### Instrument development

We modified the abstraction instrument from a prior UHC PRF benchmarking project [29] for use in this study via the REDCap™ electronic platform. We previously published our study instrument as an online supplement [20]. A condensed table of definitions of comorbidities, risk factors, and outcome variables can be found in the online supplement (Additional file 5, Table S5). The instrument contains information on demographics, pre-existing comorbidities, preoperative laboratory and radiographic test results, procedures and diagnoses, length of stay, intra- and perioperative care (e.g., ventilator settings, fluid intake and output, and medication administration), and discharge disposition. We used methodology developed by Vizient, Inc., to determine total costs of care for each encounter, inclusive of direct and indirect costs, from charge data [30].

### Data collection

The entire health record for each sampled hospitalization was reviewed, and data were manually extracted and entered into a REDCap™ database. Five abstractors participated in data collection and the first author validated the data abstraction of 100% of the records. Interrater reliability was not explicitly measured, but disagreements after the initial training period were rare.

### Statistical analysis

We performed conditional logistic regression to assess potential risk factors for L-PRF. We calculated unadjusted odds ratios (ORs) and 95% confidence intervals (CIs). We assessed all predictors supported by prior studies and predictors with *p* < 0.20 for collinearity and excluded all variables with a variance inflation factor ≥ 2.5 from consideration for multivariable analysis [31].

We developed multivariable conditional logistic regression models using purposeful variable selection [32] and a 10% change-in-estimate procedure [33] to determine if the potential for confounding was present and warranted adjustment. For final model selection, we relied on Akaike’s Information Criteria (AIC) and Bayesian Information Criteria (BIC) to identify best model fit [34]. We calculated adjusted odds ratios and 95% confidence intervals, using Stata MP® version 15.1 for all analyses. We then performed Poisson regression to analyze differences in hospital and intensive care unit length of stay between L-PRF cases and non-cases and between E-PRF cases and L-PRF cases. We used linear regression to analyze differences in total cost.

## Results

### Matched case-control sample (L-PRF and no-PRF)

There were 95 confirmed cases of L-PRF among 59,073 eligible discharges from the five sites for an overall rate of 1.61 per 1000 discharges. After 1:1 matching of L-PRF to No-PRF, our total sample (*n* = 190) was majority female, white, non-Hispanic, and covered by a third-party payer (Table 1). Most patients (59%) were 60 years of age or older. The baseline acuity of most patients was high: 72.1% had an American Society of Anesthesiologists (ASA) class of III or greater; 63.2% had two or more pre-existing comorbid conditions on admission.

**Table 1:** Patient-level Characteristics: Late Postoperative Respiratory Failure compared to No Postoperative Respiratory Failure

Variable^**a**^	Total Sample (***n*** = 190)	No PRF (***n*** = 95)	L-PRF (***n*** = 95)	Unadjusted Odds Ratio (95% CI)
**Patient Demographics**				
Age (in years), median (IQR)	63 (56, 70)	63 (55, 70)	63 (56, 70)	n/a (matching variable)
Male gender, n (%)	89 (46.8)	42 (44.2)	47 (49.5)	1.26 (0.69–2.30)
Race, n (%)				
White	139 (73.2)	71 (74.7)	68 (71.6)	referent
Black	10 (5.3)	5 (5.3)	5 (5.3)	1.01 (0.29–3.48)
Asian	20 (10.5)	9 (9.5)	11 (11.6)	1.25 (0.50–3.09)
Other	21 (11.1)	10 (10.5)	11 (11.6)	1.15 (0.43–3.11)
Primary Payer Category, n (%)				
Medicare	93 (49.0)	42 (44.2)	51 (53.7)	1.45 (0.72–2.90)
Medicaid	30 (15.8)	18 (18.9)	12 (12.6)	0.77 (0.33–1.78)
Private/ Commercial Insurance (PPO, HMO, Military)	67 (35.3)	35 (36.8)	32 (33.7)	referent
**Patient Comorbidities**				
Body Mass Index, median (IQR)	25.5 (22.7, 30.1)	25.8 (22.9, 30.2)	25.1 (22.5, 30.1)	0.99 (0.96–1.04)
ASA Class^b^ (III and above)	137 (72.9)	62 (65.3)	75 (78.9)	1.89 (1.01–3.54)
Alcohol (current drinker)	44 (23.2)	20 (21.5)	24 (25.3)	1.33 (0.63–2.80)
Asthma, n (%)	14 (7.4)	9 (9.5)	5 (5.3)	0.56 (0.19–1.68)
Chronic Kidney Disease, n (%)	17 (9.0)	7 (7.4)	10 (10.5)	1.50 (0.53–4.21)
Chronic Obstructive Pulmonary Disease, n (%)	13 (6.8)	5 (5.3)	8 (8.4)	1.62 (0.52–5.05)
Cardiac^c^, n (%)	37 (19.5)	12 (12.6)	25 (26.3)	2.05 (1.14–5.48)
Diabetes, n (%)	34 (17.9)	16 (16.8)	18 (19)	1.15 (0.55–2.42)
Gastroesophageal Reflux Disease, n (%)	48 (25.3)	21 (22.1)	27 (28.4)	1.36 (0.72–2.57)
Hypertension, n (%)	85 (44.7)	39 (41.1)	46 (48.4)	1.38 (0.76–2.50)
Liver Disease, n (%)	27 (14.2)	13 (13.7)	14 (14.7)	1.12 (0.45–2.79)
Neurologic Disease^d^, n (%)	39 (20.5)	12 (12.6)	27 (28.4)	3.30 (1.41–7.74)
Obstructive Sleep Apnea, n (%)	20 (10.5)	8 (8.4)	12 (12.6)	1.54 (0.61–3.88)
Smoking (current), n (%)	25 (13.2)	11 (11.6)	14 (14.7)	1.39 (0.55–3.52)
Total^e^ number of comorbid conditions at admission, mean (SD)	2.2 (1.6)	1.8 (1.5)	2.5 (1.5)	1.39 (1.12–1.73)
**Preoperative Laboratory Tests**				
Albumin < 3.5 g/dL	23 (12.1)	5 (53)	18 (19)	4.66 (1.52–14.27)

^a^Refer to Supplemental Table 1. Definitions of Comorbidities, Predictors, and Primary Outcome Variables

^b^American Society of Anesthesiologists (ASA) Classification System

^c^Cardiovascular disease: Includes heart attack, myocardial infarction, STEMI (ST elevation acute myocardial infarction), NSTEMI (non-ST elevation acute myocardial infarction), angina, dysrhythmia, valve disease (mitral, aortic), cardiomyopathy

^d^Neurologic disease: includes disease/deficit such as spinal cord injury, paralysis (e.g., following stroke or trauma), stroke, Parkinson’s, Cerebral Palsy, traumatic brain injury, hypoxic or anoxic brain injury

^e^Comorbid conditions included in this total: alcohol use, asthma, chronic kidney disease, chronic obstructive pulmonary disease, cardiac disease, dementia, diabetes (treated with oral or injectable antihyperglycemic agents), dysphagia, dyspnea (on admission at rest or with exertion), functional status (partially or wholly dependent,) gastroesophageal reflux disease, heart failure, home continuous positive airway pressure (CPAP) use, home oxygen use, hypertension, impaired sensorium (acutely confused or delirious), liver disease, neurologic disease, obstructive sleep apnea, respiratory infection (current), sepsis (present on admission), smoking, weight loss (> 10% unplanned in previous 3 months). OR is per each additional comorbidity

Among the matched cohort, most patients had general anesthesia (*n* = 186, 97.9%) and received a neuromuscular blocking agent (*n* = 178, 93.7%) (Table 2). The most common neuromuscular blocking agent used for induction was rocuronium (*n* = 134, 70.9%); the next most common was vecuronium (n = 17, 9.0%). Surgery most often involved open procedures of the abdomen or pelvis (*n* = 122, 64.6%), followed by open procedures of the head and neck (*n* = 21, 11.1%). Most patients received a benzodiazepine (*n* = 133, 70.0%) and almost all patients (*n* = 181, 95.3%) received an opioid. Ventilator tidal volume ranged from a median low of 6.8 cc/kg for predicted body weight (IQR 6.1, 7.7) to a median high of 8.8 cc/kg for predicted body weight (IQR 7.9, 9.9). Most patients had a positive fluid balance at the end of the operating room procedure (*n* = 183, 96.3%) and 24 hours after the operating room procedure (*n* = 167, 87.9%).

**Table 2:** Procedure-level Characteristics: Late Postoperative Respiratory Failure compared to No Postoperative Respiratory Failure

Variable^**a**^	Total Sample (***n*** = 190)	No PRF (***n*** = 95)	L-PRF (***n*** = 95)	Unadjusted Odds Ratio (95% CI)
**Procedure**				
General Anesthesia, n (%)	186 (97.9)	92 (96.8)	94 (98.9)	3.67 (0.32–41.78)
Neuromuscular Blockade, n (%)	178 (93.7)	87 (91.6)	91 (95.8)	2.33 (0.60–9.02)
Anesthesia duration per hour, median (IQR)	6.4 (4.4, 8.1)	5.5 (3.9, 7.1)	7.1 (4.7, 8.9)	1.23 (1.09–1.41)
Surgical duration per hour, median (IQR)	4.6 (3.0, 6.5)	3.8 (2.7, 5.6)	5.3 (3.3, 7.2)	1.21 (1.07–1.39)
**Intraoperative Ventilator Management**				
Tidal Volume (highest) (cc/kg) predicted body weight, median (IQR)	8.8 (7.9, 9.9)	8.8 (7.9, 9.9)	8.9 (7.8, 10.3)	1.13 (0.95–1.34)
Positive End Expiratory Pressure (highest), median (IQR)	5 (5, 6)	5 (5, 6)	5 (5, 8)	1.09 (0.95–1.26)
Peak Inspiratory Pressure (highest), median (IQR)	23 (19, 28)	21 (18, 25.5)	25 (20, 29)	1.14 (1.07–1.21)
**Intraoperative Fluid Management**				
Crystalloid Administered (per 1000 cc), median (IQR)	2.2 (1.5, 3.2)	2 (1.4, 3)	2.5 (1.6, 3.5)	1.24 (0.99–1.55)
Colloid Administered (per 250 cc), median (IQR)	0 (0, 4)	0 (0, 2)	1 (0, 4)	1.10 (1.01–1.20)
Blood Transfused (per 250 cc), median (IQR)	0 (0, 1)	0 (0, 0)	0 (0, 2)	1.02 (0.99–1.05)
Net Fluid in first 24 hours post-op (cc/kg), median (IQR)	24.4 (8.6, 47.0)	21.2 (7.9, 37.8)	26.6 (9.2, 65.5)	1.007 (1.0003–1.01)

^a^Refer to Additional file 5, Table S5. Definitions of Comorbidities, Risk Factors, and Outcome Variables

The etiology of L-PRF was primarily of pulmonary origin (e.g., aspiration pneumonitis, pneumonia) in 58 cases (61.0%) and of extrapulmonary in origin (e.g., sepsis, postoperative hemorrhage) in 37 cases (39.0%) (Additional file 6, Table S6). The most common etiologies were infectious in nature (*n* = 38, 40.0%) (e.g., sepsis, pneumonia).

### Outcomes: L-PRF versus no-PRF

Cases of L-PRF had higher unadjusted in-hospital mortality, hospital and intensive care unit (ICU) length of stay, and total costs (Table 3). Compared to cases of No-PRF, and adjusting for age, hospital, procedure, ASA class, and total number of comorbidities, the average hospital length of stay for cases of L-PRF was 5.03 (95% CI 4.01–6.31) times as long, the average ICU length of stay was 21.99 (95% CI 11.76–41.11) times as long, and the average total cost was $166 K greater (95% CI $131 K - $202 K). Compared to No-PRF, cases of L-PRF were less often discharged home able to care for themselves and more often discharged to a long-term care facility or a skilled nursing facility.

**Table 3:** Outcomes: Patients with Late PRF compared to Patients with No PRF and Patients with Early PRF

Variable	No PRF (***n*** = 95)	E-PRF (***n*** = 319)	L-PRF (***n*** = 95)	No PRF v L-PRF Point Estimate (95% CI)	E-PRF v. L-PRF Point Estimate (95% CI)
Death in Hospital, n (%)	0 (0)	44 (13.8)	30 (31.6)	n/a	2.88 (1.69–4.94)^a, c^
Hospital Length of Stay (days), median (IQR)	5 (4, 8)	13 (7, 23)	33 (20, 40)	5.03 (4.01–6.31) ^b, d^	2.30 (1.89–2.79) ^b, d^
ICU Length of Stay (days), median (IQR)	0 (0, 1)	3 (1, 11)	13 (1, 29)	21.99 (11.76–41.11) ^b, d^	2.28 (1.81–2.88) ^b, d^
Total Cost (dollars), median (IQR)	24,892 (16,210, 38,033)	79,755 (47,287, 140,000)	150,532 (102,953, 238,117)	166,000 (131,000 – 202,000)^b, e^	99,952 (61,532 – 138,372) ^b, e^
Discharge Status Home independent v all other discharge dispositions (LTAC, SNF, Rehab, another Hospital, Hospice), n(%)^f^	60 (63.2)	68 (21.3)	7 (7.4)	74.3 (10.12–546.01)^a, c^	3.41 (1.51, 7.69)^a, c^

^a^Unadjusted odds ratio

^b^Adjusted for age, hospital, procedure, ASA classification, and total number of comorbidities

^c^Logistic regression, expressed as the odds ratio

^d^Poisson regression, expressed as the incident rate ratio (IRR)

^e^Linear regression, expressed as the difference between groups in units being analyzed (e.g., dollars)

^f^*LTAC* long term acute care facility, *SNF* skilled nursing facility, *Rehab* rehabilitation facility, either a stand-alone location or a separate wing of the original hospital

### Outcomes: E-PRF versus L-PRF

The median postoperative day of diagnosis for E-PRF was 1 (IQR 0, 2; Range 0–5) and for L-PRF was 10 (IQR 7, 15; Range 6–57). Compared to cases of E-PRF, cases of L-PRF had higher unadjusted in-hospital mortality, hospital length of stay, ICU length of stay, and total costs (Table 3). Compared to E-PRF, and adjusting for age, hospital, procedure, ASA class, and total number of comorbidities, the average hospital length of stay of cases of L-PRF was 2.30 (95% CI 1.89–2.79) times as long, the average ICU length of stay was 2.28 (95% CI 1.81–2.88) times as long, and the average total cost was $99,952 greater (95% CI $61,532 - $138,372). Compared to E-PRF, cases of L-PRF were less often discharged home able to care for themselves and more often discharged to a long-term care facility or a skilled nursing facility.

Relative to E-PRF, L-PRF was associated with increased odds (OR 2.2, 95% CI 1.66–2.97) of having another adverse, non-mortality patient safety event. L-PRF was associated with increased odds of perioperative hemorrhage or hematoma (OR 4.05, 95% CI 1.66–9.85) and postoperative acute kidney injury requiring dialysis (OR 6.55, 95% CI 2.97–14.43). The concept of “cascade iatrogenesis”, the sequential development of additional medical complications following a seemingly harmless first event, such as analgesia for postoperative pain, has been described in terms of nursing care for older adults who develop PRF [35–37] and may be applicable here as well. We did not find an association with any other patient safety events or hospital acquired infections (e.g., perioperative sepsis, surgical site infection).

### Risk factors: L-PRF versus no PRF

Among patient-related factors, ASA class of III or greater, pre-existing cardiac disease, pre-existing neurologic disease, higher number of pre-existing comorbidities, and low albumin were associated with increased unadjusted odds of L-PRF (Table 1). Procedure-related factors associated with increased unadjusted odds of L-PRF included: longer duration of anesthesia and surgery; higher peak inspiratory pressure; higher intra-operative volume of infused colloid; receiving blood in the operating room; increased intravenous fluid intake in the operating room and at 24 hours postop; and 24-hour net positive fluid balance (Table 2).

### Multivariable analysis

In the final multivariable conditional logistic regression model, after matching cases with L-PRF 1:1 to cases with No-PRF based on age, hospital, and surgical procedure, factors associated with L-PRF included: the presence of neurologic disease at admission, anesthesia duration, and maximum peak inspiratory pressure (Table 4).

**Table 4:** Multivariable Conditional Logistic Regression Model of Risk Factors Associated with Late Postoperative Respiratory Failure

Variable	Adjusted Odds Ratio (95% Confidence Interval)
Neurologic Disease (present on admission)^a^	4.36 (1.81–10.46)
Anesthesia Duration (per hour)	1.22 (1.04–1.44)
Maximum Operative Peak Inspiratory Pressure (per cm water)	1.14 (1.06–1.22)

^a^Neurologic disease: includes disease/deficit such as spinal cord injury, paralysis (e.g., following stroke or trauma), stroke, Parkinson’s, Cerebral Palsy, traumatic brain injury, hypoxic or anoxic brain injury

## Discussion

We compared outcomes for patients who developed L-PRF to patients who did not develop PRF (No-PRF) and to patients who developed E-PRF. L-PRF was associated with increased morbidity, mortality, hospital and ICU length of stay, and total costs when compared to both No-PRF and E-PRF. Cases of L-PRF were often of infectious etiology (e.g., sepsis, pneumonia), with a smaller proportion due to surgical or hospital complications (e.g., acute vascular insufficiency of the intestine, postoperative hemorrhage). These findings align with those described by Moore et al. [38], who found age older than 60 years and presence of any comorbidity to be major risk factors for sepsis, and Chughati et al. [39], who found hospital-acquired and ventilator-associated pneumonia continue to be common complications after surgery, despite recent emphasis on ICU prevention bundles, with non-modifiable risk factors inclusive of age and preoperative functional ability. The incidence of infectious complications emphasizes the need for early recognition of at-risk patients.

While the impact of PRF (e.g., length of stay and cost) have been detailed in two large, multisite studies set in Academic Medical Centers [9] and the Veteran’s Administration, [40] we have quantified, using newer data, the significantly worse outcomes associated with L-PRF when compared to E-PRF and No-PRF. We also assessed for risk factors associated with L-PRF, when compared to a matched cohort of patients with No-PRF, and identified pre-existing neurologic disease, increased anesthesia duration, and increased maximum peak inspiratory pressure as significant and potentially modifiable risk factors. These findings add to a growing body of critical care and surgical literature examining factors associated with PRF.

### Intraoperative peak inspiratory pressure

A key finding from our study is that a higher maximum intraoperative peak inspiratory pressure (PIP) was associated with increased odds of developing L-PRF. It has long been accepted that changes in the respiratory system occur as soon as general anesthesia and MV are initiated [41, 42]; it has more recently been postulated that these changes can last several days [43] to several weeks [44–47]. Less clear is which components of MV are most harmful or protective in existing ARDS as well as in healthy lungs when the intent is to reduce the risk of ventilator induced lung injury.

In a 29-center study of 2466 moderate and severe ARDS patients, [48] there was substantial center-to-center variability in early adherence to lung protective ventilation (LPV) (0–65%) and mortality (16.7–73.3%). Center standardized mortality rates (SMRs), which calculated the ratio between observed and expected mortality, ranged from 0.33–1.98 and, of the treatment-level factors explored, only early LPV was associated with lower SMR. The authors concluded that early adherence to LPV was associated with lower center mortality and postulated LPV to be a surrogate for overall quality of care processes. The authors defined LPV as tidal volume < 6.5 ml/kg of predicted body weight (PBW) and plateau pressure and/or peak inspiratory pressure (PIP) < 30 cmH_2_O. In a case-control study of 50,367 surgical hospitalizations, with 93 (0.2%) cases of postoperative ARDS, the authors found higher median PIP in ARDS patients (27 versus 21, *p* < 0.001) and stated their data suggest intraoperative exposure to elevated PIP with a lack of positive end expiratory pressure (PEEP) was associated with the development of postoperative ARDS, possibly due to barotrauma and atelectrauma produced by these ventilator settings [11]. While they recommended further investigation, the authors concluded their findings potentially offered clinicians opportunities to reduce postoperative ARDS. These two studies by Qadir et al. and Blum et al. are relevant to our work as the authors deemed low PIP to be an acceptable surrogate of LPV in the absence of information about plateau pressures, which they found to be measured only 49.6% of the time on day one in the ICU [48] and not collected at all in the intraoperative environment [11]. While we would have liked to evaluate plateau pressures, these data were not documented in the operating suite for adults undergoing elective surgical procedures during the timeframe for our study. Because, in many intraoperative situations, LPV is not always verified with a plateau pressure, we felt our use of PIP was a reasonable surrogate measure of adherence to an LPV approach.

While plateau or driving pressures may best correlate with lung injury, evidence of an association between PIP and hospital mortality from ARDS and acute hypoxic respiratory failure is also provided by the international, multi-site LUNG SAFE study [49]. In 2377 patients enrolled in LUNG SAFE, potentially modifiable factors associated with increased hospital mortality on multivariable analyses included lower PEEP; higher PIP, plateau pressure, and driving pressure; and increased respiratory rate. In an invited editorial of the LUNG SAFE study, the authors further reinforced the conclusion that PIP was higher in non-survivors [50]. They concluded that PIP is a potential target for improvement of outcomes in ARDS patients.

The Qadir et al. findings of low adherence to LPV in known ARDS patients is interesting considering decades of evidence that LPV improves survival in ARDS, [51] but perhaps makes more sense when we consider the evolution of LPV from an initial focus on lower tidal volume and higher PEEP with or without lung recruitment measures, [52–54] to the addition of lower plateau pressures, [55] to a more recent focus on lower driving pressure (the difference between plateau pressure and PEEP) [56]. More recently, literature has emerged on dynamic, rather than static, indicators of energy load (e.g., flow amplitude and the clinician-selected flow waveform), [57–59] Future research to evaluate applicability in the operating suite ventilator management of adult elective surgery patients and possible associations between these modifiable determinants of ventilator induced lung injury (VILI) and PRF may be warranted.

Our finding of an association between increased intraoperative PIP and L-PRF suggests more research is needed to assess the long-term effects of anesthesia and intraoperative LPV on PRF. Specifically, future research to assess possible associations between measures of mechanical power delivered to healthy lungs and development of PRF are needed. Several publications describe the physiologic changes to the respiratory system (e.g., alteration in respiratory muscle function, modification in respiratory mechanics, and reduction in lung volumes, factors which lead to increased atelectasis, decreased functional residual capacity, and decreased vital capacity) that occur upon induction of general anesthesia and initiation of mechanical ventilation [41, 42] and that can persist for several days [43] to several weeks [44–47]. These physiologic changes, combined with the published theory of cascade iatrogenesis in adverse events, [36] to include postoperative respiratory failure, [37] and the known injurious effects of barotrauma and atelectrauma, further highlight the need for future research to explore the association of intraoperative LPV, to include PIP, and the development of L-PRF.

### Anesthesia duration

Our findings align with prior studies regarding increased risk of L-PRF with increased duration of anesthesia and surgery [60]. While one study identified risk factors for six common procedures (pancreatectomy, hepatectomy, esophagectomy, abdominal aortic aneurysm repair, open aortoiliac repair, and lung resection) and found the risk factors varied by procedure type, the one risk factor that was consistent across all procedures was prolonged procedure time [61]. Aside from consideration of less aggressive operative approaches and optimization of perioperative supplies, team staffing and expertise, there are limited options available to reduce total surgery and anesthesia duration. While we did not find an association between type of anesthesia and L-PRF, very few patients in our cohort received conscious sedation, also known as monitored anesthesia care. In abdominal aortic aneurysm and aortoiliac repair, an endovascular approach using monitored anesthesia care was associated with lower risk of PPCs (OR 0.48, 95% CI 0.24–0.92) [62].

### Pre-existing neurologic comorbidities

We also found an association between pre-existing neurologic comorbidities and increased odds of L-PRF. While pre-existing neurologic comorbidities are largely non-modifiable, [7] we believe that—given the mortality, morbidity, and costs associated with PRF—more research is needed to determine if some neurologic disorders might be responsive to preoperative optimization. Examples of potential interventions include protocolized swallow evaluation and swallow training to reduce aspiration risk, incentive spirometry to reduce atelectasis, and exercise programs to improve strength in anticipation of early postoperative mobilization. Some studies on colorectal and cardiac surgery patients have demonstrated positive benefits of multimodal “prehabilitation” to optimize such factors as nutrition, exercise, and smoking cessation [63–65]. Delay of elective procedures to better prehabilitate the patient might also be considered in high-risk neurological patient populations. In addition, a better understanding and awareness of which neurologic diseases are at highest risk would allow providers to target these patients for prevention in the perioperative period.

### Limitations

Our results should be interpreted with some caution. These findings are based on retrospective analysis of a rare event in five academic medical centers within one health system, for the years 2012–2015, a timeframe during which the UC systems were in the process of implementing perioperative bundles such as Enhanced Recovery After Surgery (ERAS) [66]. These interventions might account for the limited variability we saw between groups with some variables of interest, such as mechanical ventilation tidal volume. We were unable to analyze some variables of interest, such as operative plateau pressure and minute-to-minute documentation of all ventilator settings due to missing documentation and limited resources for manual data abstraction. Lung protective lung ventilation involves multiple ventilator parameters, their interaction with the patient’s physiology, and surgical factors (e.g., laparoscopic surgery with pneumoperitoneum, Trendelenburg position, body habitus) [67]. The rare nature of PRF also makes analysis complex. While the OR and CIs for preexisting neurologic conditions were both high and wide, respectively, due to the low incidence (*n* = 27 in the L-PRF group and *n* = 12 in the No-PRF group), the ORs and CIs for anesthesia duration and PIP were reasonable and plausible. We were also unable to analyze the effect of preoperative frailty [68], which has been shown to be associated with significant morbidity and mortality, [69] and other comorbidities of interest due to the incidence of PRF as a rare event. The ability to collect these data is crucial for future studies. These study limitations are similar to those described by Blum et al., in their case-control study of 93 surgical patients who developed postoperative ARDS [11] and Chen et al., in their case-control study of 36 patients who required unplanned reintubation following general anesthesia [70]. Despite the limitations associated with studying rare events, early and novel studies on a topic, such as our study of L-PRF, are often hypothesis generating and serve as a good launching point to design higher powered future studies.

Late PRF is associated with significant morbidity, mortality, and increased hospital and intensive care unit length of stay and healthcare costs, making it of interest to health care clinicians, administrators, and consumers/patients. A strength of our study is our database, which included 414 confirmed cases of PRF, 319 E-PRF and 95 L-PRF, among nearly 60,000 elective surgical admissions over 4 years from five academic medical centers. We believe our analysis is one of the first to describe the different risk factors and outcomes associated with E-PRF versus L-PRF.

### Future research

While the importance of studying rare adverse events to better understand modifiable risk factors is paramount to improve patient outcomes, this can only be achieved with increased power. Increasing sample size is the most effective way to improve power but is dependent upon funding for multi-center studies in which each center provides complete data for analysis. Larger, multi-center studies also allow for adjustment for other risk factors, therefore reducing variation and increasing power. Machine learning techniques (e.g., Classification and Regression Tree [CART] and random forest models) can help in identifying risk factors and building strong models. Emerging synthetic health data generation methods may also help accelerate rare event outcomes research. The UC3RC is exploring all of these avenues. We continue to add to our database and work to form collaborative relationships with other leading clinical research institutions to increase sample size and statistical power. We are currently transitioning our data abstraction, curation, and validation methods from manual to automated and mapping the data to standardized taxonomies to better facilitate multi-center collaborations. We also continue to seek additional resources to expand our effort.

Future studies of rare events should consider a composite comorbidity index (e.g., Charlson and/or Elixhauser) rather than a total number of comorbidities. As risk for suboptimal outcomes often changes during the hospitalization, studies should also consider scores like the Sequential Organ Failure Assessment (SOFA) and/or Acute Physiology, Age and Chronic Health Evaluation (APACHE) scores. Analysis of frailty on surgical outcomes is also needed to determine if some elective procedures should be deferred entirely.

While manual data abstraction by trained clinicians has many benefits, it is costly and time-consuming and limits the scope of this type of research. As electronic health records continue to evolve and clinician documentation is increasingly mapped to standardized taxonomies (e.g., SNOMED CT, RxNorm, LOINC), the burden of abstraction will become less prohibitive. While automatic data capture from the electronic health record may prove beneficial for future studies, it must be balanced against burden of validation of the documentation and mapping to the existing standardized taxonomies.

## Conclusions

Prevention of PRF is of great importance as PRF is a source of high morbidity and mortality. We have identified, in a consecutive sample of nearly 60,000 elective surgical discharges, three potentially modifiable risk factors for L-PRF: 1) intraoperative peak inspiratory pressure; 2) duration of anesthesia; and 3) pre-existing neurological disease. Identification of these factors should be used to create and investigate targeted interventions aimed at reducing L-PRF. We propose that these interventions might include a reduction of peak inspiratory pressures as a component of a lung protective ventilation, resulting in a personalized anesthesia approach to each patient’s physiologic response. Second, institutional focus on optimization of operating room flow to reduce duration of anesthesia and surgery through enhanced staffing and supplies may be considered. Lastly, identification of sub-populations of patients with pre-existing neurologic disease most apt to benefit from targeted perioperative pre-habilitation. Ongoing research is needed to better understand these risk factors and to develop and validate interventions for prevention of L-PRF.

## Supplementary Information

**Additional file 1 Table S1.** Distribution of Age (by Decade) Used in Matching Process. Distribution of age (by decade) used in matching of case-control pairs.

**Additional file 2 Table S2.** Distribution of Hospital Site Used in Matching Process. Distribution of hospital site used in matching of case-control pairs.

**Additional file 3 Table S3.** Distribution of Surgical Procedure (by Body Organ or System) Used in Matching Process. Distribution of surgical procedure (by body organ or system) used in matching of case-control pairs.

**Additional file 4 Table S4.** Distribution of Surgical Procedure (by Modified Clinical Classification) Used in Matching Process. Distribution of surgical procedure (by modified clinical classification group) used in matching of case-control pairs.

**Additional file 5 Table S5.** Definitions of Comorbidities, Risk Factors, and Outcome Variables. Definitions of comorbidities, risk factors, and outcome variables used in analysis of case-control pairs.

**Additional file 6 Table S6.** Distribution of Most Likely Etiology of L-PRF. Distribution of most likely etiology of L-PRF, by total cohort, pulmonary subset, and extrapulmonary subset.

## Data Availability

The datasets used and/or analyzed during the current study are available from the corresponding author on reasonable request.

## Abbreviations

ALI: Acute Lung Injury
ARDS: Acute Respiratory Distress Syndrome
AHRQ: Agency for Healthcare Research and Quality
ASA: American Society of Anesthesiologists
CI: Confidence Interval
E-PRF: Early Postoperative Respiratory Failure
ICU: Intensive Care Unit
IQR: Interquartile Range
L-PRF: Late Postoperative Respiratory Failure
LPV: Lung Protective Ventilation
MV: Mechanical Ventilation
No-PRF: No Postoperative Respiratory Failure
OR: Odds Ratio
PSI 11: Patient Safety Indicator 11
PIP: Peak Inspiratory Pressure
PEEP: Positive End Expiratory Pressure
PPCs: Postoperative Pulmonary Complications
PRF: Postoperative Respiratory Failure
UHC: University Healthsystem Consortium
UC3RC: University of California Critical Care Research Collaborative
