# Supplementary material for: Outcomes and risk factors for delayed-onset postoperative respiratory failure: a multi-center case-control study by the University of California Critical Care Research Collaborative (UC3RC)
Source: BMC Anesthesiol. 2022 May 14;22:146. doi: 10.1186/s12871-022-01681-x (PMC9107656; doi:10.1186/s12871-022-01681-x)
Supplement: Supplementary file 1 — Additional file 1 Table S1. Distribution of Age (by Decade) Used in Matching Process. Distribution of age (by decade) used in matching of case-control pairs. [file 12871_2022_1681_MOESM1_ESM.docx]

**Additional File 1**

**eTable1: Distribution of Age (by Decade) Used in Matching Process**

| **Age (by decade)** | **Number of Case-Control Pairs,**  **n (%)** |
| --- | --- |
| 1 (18-19) | 1 (1.1) |
| 2 (20-29) | 2 (2.1) |
| 3 (30-39) | 3 (3.2) |
| 4 (40-49) | 8 (8.4) |
| 5 (50-59) | 25 (26.3) |
| 6 (60-69) | 32 (33.7) |
| 7 (70-79) | 18 (18.9) |
| 8 (80-89) | 6 (6.3) |
| 9 (90-99) | 0 (0) |
| Total | 95 (100) |
