# Supplementary material for: Outcomes and risk factors for delayed-onset postoperative respiratory failure: a multi-center case-control study by the University of California Critical Care Research Collaborative (UC3RC)
Source: BMC Anesthesiol. 2022 May 14;22:146. doi: 10.1186/s12871-022-01681-x (PMC9107656; doi:10.1186/s12871-022-01681-x)
Supplement: Supplementary file 2 — Additional file 2 Table S2. Distribution of Hospital Site Used in Matching Process. Distribution of hospital site used in matching of case-control pairs. [file 12871_2022_1681_MOESM2_ESM.docx]

**Additional File 2**

**eTable2: Distribution of Hospital Site Used in Matching Process**

| **Hospital Site, n (%)** | **Number of Case-Control Pairs,**  **n (%)** |
| --- | --- |
| Site 1 (University of California Davis) | 13 (13.7) |
| Site 2 (University of California San Francisco) | 29 (30.5) |
| Site 3 (University of California Irvine) | 16 (16.8) |
| Site 4 (University of California Los Angeles) | 20 (21.1) |
| Site 5 (University of California San Diego) | 17 (17.9) |
| Total | 95 (100) |
