# Supplementary material for: Outcomes and risk factors for delayed-onset postoperative respiratory failure: a multi-center case-control study by the University of California Critical Care Research Collaborative (UC3RC)
Source: BMC Anesthesiol. 2022 May 14;22:146. doi: 10.1186/s12871-022-01681-x (PMC9107656; doi:10.1186/s12871-022-01681-x)
Supplement: Supplementary file 3 — Additional file 3 Table S3. Distribution of Surgical Procedure (by Body Organ or System) Used in Matching Process. Distribution of surgical procedure (by body organ or system) used in matching of case-control pairs. [file 12871_2022_1681_MOESM3_ESM.docx]

**Additional File 3**

**eTable 3: Distribution of Surgical Procedure (by Body Organ or System) Used in Matching Process**

| **Surgery Region (by organ or body system)** | **Number of Case Control Pairs** |
| --- | --- |
| Digestive System | 44 |
| Musculoskeletal System | 13 |
| Nervous System | 10 |
| Vascular System | 3 |
| Urinary System | 12 |
| Female Genital Organs | 4 |
| Endocrine System | 3 |
| Miscellaneous Diagnostic and Therapeutic Procedures | 4 |
| Hemic and Lymphatic System | 2 |
| Total | 95 |
