# Supplementary material for: Outcomes and risk factors for delayed-onset postoperative respiratory failure: a multi-center case-control study by the University of California Critical Care Research Collaborative (UC3RC)
Source: BMC Anesthesiol. 2022 May 14;22:146. doi: 10.1186/s12871-022-01681-x (PMC9107656; doi:10.1186/s12871-022-01681-x)
Supplement: Supplementary file 4 — Additional file 4 Table S4. Distribution of Surgical Procedure (by Modified Clinical Classification) Used in Matching Process. Distribution of surgical procedure (by modified clinical classification group) used in matching of case-control pairs. [file 12871_2022_1681_MOESM4_ESM.docx]

**Additional File 4**

**eTable 4: Distribution of Surgical Procedure (by modified Clinical Classification) Used in Matching Process**

| **Single-level CCS, Multiple-level CCS, or Other Category** | **Relevant CCS Code** | **Description** | **Number of Case Control Pairs** |
| --- | --- | --- | --- |
| Multiple (level 3) | 1.1.3 | Other incision and excision of CNS | 8 |
| Single | 103 | Nephrotomy and nephrostomy | 1 |
| Single | 104 | Nephrectomy; partial or complete | 2 |
| Single | 105 | Kidney transplant | 1 |
| Single | 109 | Urethral repair | 1 |
| Single | 112 | Other OR therapeutic procedures of urinary tract | 7 |
| Single | 12 | Other therapeutic endocrine procedures | 3 |
| Multiple (level 3) | 12.5.1 | Total abdominal hysterectomy | 4 |
| Multiple (level 3) | 14.13.3 | Other muscle and tendon procedures | 4 |
| Other* | 14.3.5 | Fracture repair, remove hardware | 1 |
| Other* | 158.2 | Spinal fusion, dorsolumbar (lower thoracic/upper lumbar) | 2 |
| Other* | 158.3 | Spinal fusion, lumbar | 4 |
| Single | 159 | Bone biopsy | 1 |
| Single | 161 | Other OR therapeutic procedures on bone | 1 |
| Single | 176 | Other organ transplantation | 4 |
| Single | 3 | Laminectomy; excision intervertebral disc | 1 |
| Single | 56 | Other vascular bypass and shunt; not heart | 2 |
| Other** | 7.19.2.1 | Other OR procedures on blood vessels, clipping of aneurysm | 1 |
| Single | 74 | Gastrectomy | 1 |
| Single | 75 | Small bowel resection | 1 |
| Other† | 78.1 | Colorectal resection, open | 3 |
| Other† | 78.2 | Colorectal resection, laparoscopic | 1 |
| Other | 8.4.3 | Regional lymph node excision | 1 |
| Other†† | 86.2.1 | Other hernia repair, incisional, open | 2 |
| Single | 89 | Exploratory laparotomy | 2 |
| Single | 9 | Other OR therapeutic nervous system procedures | 1 |
| Multiple (level 3) | 9.16.1 | Open cholecystectomy | 1 |
| Multiple (level 3) | 9.28.1 | Closure of stoma of large intestine | 1 |
| Multiple (level 3) | 9.28.6 | Other lower GI procedures | 4 |
| Multiple (level 3) | 9.31.1 | Radical pancreaticoduodenectomy | 9 |
| Multiple (level 3) | 9.31.3 | Excision or destruction of peritoneal tissue | 3 |
| Multiple (level 3) | 9.31.5 | Other gastrointestinal therapeutic procedures | 15 |
| Single | 90 | Excision; lysis peritoneal adhesions | 2 |
| Total |  |  | 95 |

***** subdivided single-level CCS 158 because operations on different parts of the spine plausibly involve different risks for PRF

** subdivided multi-level CCS 7.19.2 to account for aneurysm clipping uniquely involving the cerebral blood vessels

† subdivided single-level CCS 78 to distinguish open from laparoscopic colectomy/proctectomy

†† subdivided single-level CCS 86 into different types of hernias, then further subdivided the incisional hernia subgroup into open vs. laparoscopic
