# Supplementary material for: Outcomes and risk factors for delayed-onset postoperative respiratory failure: a multi-center case-control study by the University of California Critical Care Research Collaborative (UC3RC)
Source: BMC Anesthesiol. 2022 May 14;22:146. doi: 10.1186/s12871-022-01681-x (PMC9107656; doi:10.1186/s12871-022-01681-x)
Supplement: Supplementary file 5 — Additional file 5 Table S5. Definitions of Comorbidities, Risk Factors, and Outcome Variables. Definitions of comorbidities, risk factors, and outcome variables used in analysis of case-control pairs. [file 12871_2022_1681_MOESM5_ESM.docx]

**Additional File 5**

**eTable 5: Definitions of Comorbidities, Risk Factors, and Outcome Variables**

| **Variable** | **Definition** |
| --- | --- |
| **Comorbidity** | presence or absence is based on documentation in the preoperative history and physical |
| Alcohol (current drinker) | Current includes drinking daily within 4 weeks of this  admission |
| ASA Class | American Society of Anesthesiologists (ASA) class |
| Asthma | asthma, reactive airway disease |
| Body mass index (BMI) | calculated as height(m) x weight(kg)-2 |
| Chronic kidney disease | CKD, chronic kidney disease with stage, end stage renal failure, renal disease, renal failure, kidney failure |
| Chronic obstructive pulmonary disease | COPD, chronic bronchitis, emphysema |
| Cardiac disease | heart attack, myocardial infarction, STEMI (ST elevation acute myocardial infarction), NSTEMI (non-ST elevation acute myocardial infarction), angina, dysrhythmia, valve disease (mitral, aortic), cardiomyopathy |
| Dementia | Alzheimer's, senile dementia, dementia |
| Diabetes | diabetes mellitus type 1 or 2 |
| Dysphagia | patient reports of difficulty swallowing |
| Dyspnea | dyspnea on admission – at rest or with exertion |
| Functional status | able to perform activities of daily living independently or needs partial or full assist |
| Gastroesophageal reflux disease | GERD |
| Heart failure | congestive heart failure, heart failure with reduced or preserved ejection fraction |
| Hypertension | high blood pressure |
| Impaired sensorium | acutely confused or delirious |
| Liver disease | cirrhosis, hepatomegaly, liver failure, ascites |
| Neurological disease | disease/deficit such as spinal cord injury, paralysis (e.g., following stroke or trauma), stroke, Parkinson's, Cerebral Palsy, traumatic brain injury, hypoxic or anoxic brain injury |
| Obstructive sleep apnea | sleep apnea, OSA |
| Predicted body weight (PBW) | calculated using MDCalc: <https://www.mdcalc.com/ideal-body-weight-adjusted-body-weight> |
| Respiratory infection | current, present on admission |
| Sepsis | Septic shock, SIRS criteria, sepsis present on admission |
| Smoking (current) | current or prior smoker (cigarettes, vaping, marijuana) |
| Weight loss | >10% in previous 3 months, unplanned |
| Total number of comorbid conditions at admission | Comorbid conditions included in this total: alcohol use, asthma, chronic kidney disease, chronic obstructive pulmonary disease, cardiac disease, dementia, diabetes (treated with oral or injectable antihyperglycemic agents), dysphagia, dyspnea (on admission at rest or with exertion), functional status (partially or wholly dependent,) gastroesophageal reflux disease, heart failure, home continuous positive airway pressure (CPAP) use, home oxygen use, hypertension, impaired sensorium (acutely confused or delirious), liver disease, neurologic disease, obstructive sleep apnea, respiratory infection (current), sepsis (present on admission), smoking, weight loss (>10% unplanned in previous 3 months). OR is per each additional comorbidity. |
| **Preoperative Diagnostic and Laboratory Tests** |  |
| Abnormal Chest Radiograph | pneumothorax, pneumonia, pulmonary edema, cardiomegaly, ARDS, consolidation, atelectasis, pleural effusion |
| Abnormal 12-lead Electrocardiogram | cardiac arrhythmias, evidence of old or new  myocardial infarction |
| **Operative Management** |  |
| Duration of surgery | incision time to closure |
| Duration of anesthesia | duration of time under care by anesthesiologist |
| Morphine equivalent units | Calculated using ClinCalc: <https://clincalc.com/opioids/> |
| Benzodiazepine equivalent units | Calculated using ClinCalc: <https://clincalc.com/benzodiazepine/> |
| **Outcome** | **Definition** |
| LOS Outlier | observed LOS is greater than the 99th percentile within the base admission MS-DRG |
