# Supplementary material for: Outcomes and risk factors for delayed-onset postoperative respiratory failure: a multi-center case-control study by the University of California Critical Care Research Collaborative (UC3RC)
Source: BMC Anesthesiol. 2022 May 14;22:146. doi: 10.1186/s12871-022-01681-x (PMC9107656; doi:10.1186/s12871-022-01681-x)
Supplement: Supplementary file 6 — Additional file 6 Table S6. Distribution of Most Likely Etiology of L-PRF. Distribution of most likely etiology of L-PRF, by total cohort, pulmonary subset, and extrapulmonary subset. [file 12871_2022_1681_MOESM6_ESM.docx]

**Additional File 6**

**eTable6: Distribution of Most Likely Etiology of L-PRF**

| **Etiology** | **Number n (%)** |
| --- | --- |
| Pulmonary Etiology | 58 (61.0% of all L-PRF cases) |
| Food/Vomit Aspiration Pneumonitis | 18 (18.9%) |
| Pneumonia | 13 (13.7%) |
| Pleural Effusion | 11 (11.6%) |
| Pulmonary Edema/Fluid Overload | 9 (9.5%) |
| Pulmonary Embolism | 5 (5.3%) |
| Pneumothorax/Tension Pneumothorax | 2 (2.1%) |
| Extrapulmonary Etiology | 37 (39.0% of all L-PRF cases) |
| Sepsis, Postoperative Infection | 25 (26.3%) |
| Acute Vascular Insufficiency of Intestine, Necrotic Gut  Post-Surgery | 4 (4.2%) |
| Postoperative Hemorrhage/Shock | 3 (3.2%) |
| Cerebral Edema, Intracranial Hemorrhage | 2 (2.1%) |
| Drug-Induced Delirium/Opioid Overdosing | 1 (1.1%) |
| Graft versus Host Disease | 1 (1.1%) |
| Acute Kidney Failure | 1 (1.1%) |
| **Total** | **95 (100)** |
